# The *CaChiVI2* Gene of *Capsicum annuum* L. Confers Resistance Against Heat Stress and Infection of *Phytophthora capsici*

**DOI:** 10.3389/fpls.2020.00219

**Published:** 2020-02-26

**Authors:** Muhammad Ali, Izhar Muhammad, Saeed ul Haq, Mukhtar Alam, Abdul Mateen Khattak, Kashif Akhtar, Hidayat Ullah, Abid Khan, Gang Lu, Zhen-Hui Gong

**Affiliations:** ^1^College of Horticulture, Northwest A&F University, Yangling, China; ^2^Department of Horticulture, Zhejiang University, Hangzhou, China; ^3^College of Agronomy, Northwest A&F University, Yangling, China; ^4^Department of Agriculture, The University of Swabi, Khyber Pakhtunkhwa, Pakistan; ^5^Department of Horticulture, The University of Agriculture, Peshawar, Khyber Pakhtunkhwa, Pakistan; ^6^Institute of Nuclear Agricultural Sciences, College of Agriculture and Biotechnology, Zhejiang University, Hangzhou, China

**Keywords:** *Arabidopsis*, *CaChiVI2*, chitin-binding protein, drought, heat, pepper, resistance

## Abstract

Extreme environmental conditions seriously affect crop growth and development, resulting in substantial reduction in yield and quality. However, chitin-binding proteins (CBP) family member *CaChiVI2* plays a crucial role in eliminating the impact of adverse environmental conditions, such as cold and salt stress. Here, for the first time it was discovered that *CaChiVI2* (Capana08g001237) gene of pepper (*Capsicum annuum* L.) had a role in resistance to heat stress and physiological processes. The full-length open-reading frame (ORF) of *CaChiVI2* (606-bp, encoding 201-amino acids), was cloned into TRV2:*CaChiVI2* vector for silencing. The *CaChiVI2* gene carries heat shock elements (HSE, AAAAAATTTC) in the upstream region, and thereby shows sensitivity to heat stress at the transcriptional level. The silencing effect of *CaChiVI2* in pepper resulted in increased susceptibility to heat and *Phytophthora capsici* infection. This was evident from the severe symptoms on leaves, the increase in superoxide (O_2_^–^) and hydrogen peroxide (H_2_O_2_) accumulation, higher malondialdehyde (MDA), relative electrolyte leakage (REL) and lower proline contents compared with control plants. Furthermore, the transcript level of other resistance responsive genes was also altered. In addition, the *CaChiIV2*-overexpression in *Arabidopsis thaliana* showed mild heat and drought stress symptoms and increased transcript level of a defense-related gene (*AtHSA32*), indicating its role in the co-regulation network of the plant. The *CaChiVI2*-overexpressed plants also showed a decrease in MDA contents and an increase in antioxidant enzyme activity and proline accumulation. In conclusion, the results suggest that *CaChiVI2* gene plays a decisive role in heat and drought stress tolerance, as well as, provides resistance against *P. capsici* by reducing the accumulation of reactive oxygen species (ROS) and modulating the expression of defense-related genes. The outcomes obtained here suggest that further studies should be conducted on plants adaptation mechanisms in variable environments.

## Introduction

*In vivo* cultivated crops inevitably suffer adverse effects of biotic (pathogens, diseases, etc.) and abiotic (temperature, heavy metals, salinity, and drought) stresses (Zhai et al., 2016; Kilasi et al., 2018). Consequently, there are heavy losses in both quality and quantity with estimated yield decreases up to 50% (Maxmen, 2013; Wang et al., 2013b). Being sessile in nature, plants face a number of unfavorable environmental conditions. Though they have evolved an array of sophisticated mechanisms to combat these stresses, the combination of stresses may adversely affect plant physiology and productivity (Obata et al., 2015). Over the period of time, plants have evolved various pathways to adapt to the changing environmental conditions in order to survive and reproduce. These have been studied extensively with considerable emphasis on individual stresses at the molecular levels (Ramegowda and Senthil-kumar, 2015). Besides, in plants undergoing stress, many proteins are denatured resulting in loss of natural functions causing serious losses in yield and quality (Li et al., 2010; Khurana et al., 2013; Zhai et al., 2017). When plants are in severe stress, they reprogramme a number of biochemical reactions. For example, reduction of proteins that can bind and disassemble the aggregations for re-folding to form native proteins and as a result avoid damages due to aggregations (Shan et al., 2007; Lambert et al., 2011; Ruibal et al., 2013; Personat et al., 2014; Li Z. et al., 2016). Additionally, the mechanism of plant adaptation is initiated by the combined effect of reactive oxygen species (ROS) and stress-induced signaling pathways, which are activated together and provide an immediate response to various external stimuli (Sewelam et al., 2016). Another elegant response to different environmental stimuli, such as high temperature, drought, and pathogen infection, is that accompanied by secondary messenger signaling pathways. These pathways are activated by the imbalance of intracellular concentrations of various molecular compounds (Ranty et al., 2016). Abscisic acid (ABA) is linked with the plants responses to biotic and abiotic stresses through regulation of stomatal apertures and up-regulation of defense-related genes (Lim et al., 2015; Sah et al., 2016). Sets of defense-related genes against heat shock and drought stress, i.e., HSP20, PR-2, and CBP gene families, are reported to have a role in effector-triggered immunity (ETI), relying on intensity and time of the interacting signaling components (Tsuda and Katagiri, 2010; Thomma et al., 2011). Among the defense-related genes, chitin-binding protein genes family contributes significantly in plants adaptability and tolerance (Hejgaard et al., 1992; Ali et al., 2018). There are some CBP-encoded enzymes, which show high response during environmental stresses such as cold and high salt concentration. Furthermore, they play their contributing role in physiological processes of plants, such as ethylene production and embryogenesis (Fukamizo et al., 2003; Hamid et al., 2013). Previously, we identified 16 putative genes of CBP family in pepper plant. The transcriptomic analysis revealed that *CaChiVI2* gene had the most remarkable motif “AAAAAATTTC.” It was heat stress responsive protein highly induced by ABA hormone (Ali et al., 2018).

Pepper (*Capsicum annuum* L.) is a profitable crop extensively used as a green vegetable, as a spice in food, and as an innate source of natural coloring (Wang et al., 2017; Baoling, 2018). Pepper is thermophilic in nature, i.e., its growth and development is susceptible to extreme temperature and water deficiency (Guo et al., 2014). Pepper crop faces a number of challenges during cultivation period, especially in the summer, when water is deficient and temperature is too high. Such conditions favor *Phytophthora* blight disease, caused by the oomycete *Phytophthora capsici*. The infection significantly inhibits pollination, fertilization, and seed setting (Erickson and Markhart, 2002; Pagamas and Nawata, 2008). The severity of the *P. capsici* infection can have a devastating effect on plant morphology and growth. These include symptoms such as damping-off, leaf browning, senescence wilting, and dwarfing that cause plant death ultimately (Hausbeck and Lamour, 2004; Sy et al., 2005; Lamour et al., 2012; Zhang H.-X. et al., 2016).

Thus, it was essential to investigate this issue. Due to lack of conclusive evidence, it was difficult to prove that *CaChiVI2* gene was potentially involved in heat tolerance, drought stress, and *P. capsici* infection. Therefore, we extended our work to characterize *CaChiVI2* in pepper plant for the mentioned purpose. The gene is known for its role in regulating stress responses. The purpose of this study was to generate a meaningful background for further research through silencing of the target gene in pepper and over-expressing in *Arabidopsis via* transgenic approaches. Moreover, we were also interested in the physiological responses and changes induced by heat stress and disease susceptibility or tolerance through inoculation with *P. capsici.*

## Materials and Methods

### Plant Growth Conditions

Pepper (*Capsicum annuum* L.) cultivar AA3 maintained in Vegetable Plant Biotechnology and Germplasm Innovation lab, Northwest A&F University-China was studied in the present work. The growth conditions for pepper seedlings were 22/18°C (day/night) temperature with a 16 h photoperiod (i.e., 16 h light and 8 h dark cycle) and 65% relative humidity. *Arabidopsis* ecotype Columbia-0 (Col-0) was also cultivated at 25/20°C (day/night) temperature and the above-mentioned photoperiod and humidity conditions for overexpression of *CaChiVI2*-gene.

### Sequence Alignment and *in silico* Analysis

The protein sequences of the chitin-binding protein family (CBP) were aligned by ClustalW^1^. The phylogenetic tree was built using iTOL^2^ (Letunic and Bork, 2016). *Cis*-regulatory elements (1500-bp upstream region) were searched by PlantCARE online server^3^ (Lescot et al., 2002). Publicly available transcriptomic data of root and leaf for pepper cultivar “Zunla” were obtained from the online server^4^, and the genomic database was generated by following Liu Z. et al. (2017) and Yu et al. (2017) method and the data were presented in line-graphs (*CaChiVI2* sequence shown in Supplementary Table S1).

### Quantitative Real-Time PCR Analysis

The total-RNA was extracted as explained in our recently published papers (Ali et al., 2018; Khan et al., 2018). The first chain was synthesized by the Primer Script^TM^ Kit (TaKaRa, Dalian, China). The iQ5.0 Bio-Rad iCycler thermocycler (Bio-Rad, Hercules, CA, United States) was used for qRT-PCR, and SYBRR Premix Ex TaqTM II (TaKaRa) was used for the reaction. Pepper ubiquitin-binding gene *CaUBI3* (Wan et al., 2011) and *Arabidopsis Atactin2* was used as a reference. Relative gene expression levels were calculated according to the comparative threshold (2^–ΔΔCT^) technique (Schmittgen and Livak, 2008; Muhammad et al., 2018). All primer pairs (Supplementary Table S2) used for qRT-PCR were designed by NCBI Primer-BLAST. The expression levels were normalized and presented the mean and standard deviation (+SD) of data were obtained from three independent biological experiments with three replicates.

### Protein Localization of *CaChiVI2*

The transient transformation technique was performed using *Nicotiana benthamiana* epidermal cells to detect the protein localization of target gene (Jin et al., 2019; Ma et al., 2019). The ORF fragment (1244-bp) of *CaChiVI2* (primers in Supplementary Table S3) was inserted into the pVBG2307 vector regulated by CaMV 35S promoter and then transformed into GV3101 (*Agrobacterium tumefaciens*). The pVBG2307:GFP vector without the *CaChiVI2* gene was used as a control. The *Agrobacterium* cells were infiltrated into 4-weeks-old tobacco plant leaves (Yu et al., 2017). In a growth chamber, agro-infiltrated plants were transferred for 2–3 days. OLYMPUS BX63 automated fluorescence microscope was used for the determination of epidermal cells (Olympus, Tokyo, Japan).

### Virus Induce Gene Silencing Assay of *CaChiVI2*

The virus induce gene silencing (VIGS) technique was performed for the knock-down of pepper *CaChiVI2* gene, following the same approach explained by Liu et al. (2016). While for the construction of TRV2:*CaChiVI2* vector, 230-bp CDS fragment using the specific primer pair (Supplementary Table S4) of *CaChiVI2* gene with restriction enzymes sites *Eco*RI and *Xho*I was amplified through PCR. *CaChiVI2* was cloned into the TRV2 vector, while TRV2:00 vector was used as a negative control, whereas the TRV2:*CaPDS* (phytoene desaturase gene) was used as a positive control. Subsequently, the vector was used to transform into an *Agrobacterium tumefaciens* strain (GV3101) using the freeze-thaw method (Wang et al., 2013c). Consequently, the TRV2:00, TRV2:*CaPDS* and TRV2:*CaChiVI2* were activated with OD_600_ = 1.0. Afterward, the suspensions were infiltrated into the fully extended cotyledons leaves of pepper plants by using a 1.0 mL sterilized needleless syringe (Li et al., 2014; Feng et al., 2019). The infiltrated pepper plants were kept at 18–22°C in the growth chamber, maintaining the 16h/8h light/dark photoperiod as mentioned by Wang et al. (2013a). The leaf samples of the control and *CaChiVI2*-silenced plants were collected after 45 days when the TRV2:*CaPDS* injected leaves exhibited photo-bleaching phenotype and the silencing efficiency was measured by qRT-PCR. For the precision of results, the experiment was performed with three independent biological replicates.

### Generation of *CaChiVI2* Overexpressed *Arabidopsis* Lines

The full-length of *CaChiVI2* ORF fragment was cloned by cutting with restriction enzymes *Xba*I and *Kpn*I. The amplified product from cDNA was recovered through a gene-specific primer pair (Supplementary Table S5) and further transferred into a pVBG2307 expression vector. For overexpression analysis, recombinant fusion vector was used to transform into *Arabidopsis* plants (ecotype Columbia-0, Col-0) *via Agrobacterium tumefaciens* strain GV3101 (Clough and Bent, 1998; Guo et al., 2014). Positive transgenic lines were selected on MS-medium containing 50 mmol/L kanamycin. The transgenic lines were further grown for homozygosity till T_3_ generation. The T_3_ seeds were used for further experimental treatment.

### *P. capsici* Preparation and Inoculation

*Phytophthora capsici* (PC strain) was obtained from our laboratory and the technique used for its inoculation was similar as described in our previous studies (Zhang et al., 2018; Ali et al., 2019). The roots were sampled at different days of interval (0, 1, 2, 4, and 8 dpi), immediately frozen in liquid nitrogen and stored at −80°C for further study. The authentication of data was checked by a repeat of three times for each treatment.

### Stress Treatments and Samples Collection

To examine the transcript pattern of CBP family genes, pepper seedlings (8-weeks-old) at the stage of six to eight true leaves were used for basic thermo-tolerance treatment. Pepper seedlings were incubated at 45°C and samples were collected at 0, 1, 3, 6, 12, and 24 h post-treatment. *CaChiVI2*-silenced and TRV2:00 (control) plants were used to analyze the functions of *CaChiVI2* under heat and *P. capsici* inoculation. The seedlings were exposed to 45°C for heat stress, and samples were collected at different intervals of 0, 0.5, 1, 3, 6, 12, and 24 h. During stress treatment, seedlings were watered to avoid drought stress. Heat treated pepper plant leaves were collected for the validation of MDA content, total chlorophyll content and relative electrolyte leakage (REL) analysis at 0, 12 and 24 h.

The *CaChiVI2*-overexpressed *Arabidopsis* lines (OE2, OE3, OE5, OE6, and OE7) were selected. T_3_ homozygous lines (8-days old seedlings) grown on MS-media were incubated at 45°C to give heat stress for a period of 2 h. The seedlings were then shifted to growth chamber (22°C) for 7 days to recover and the survival rate was recorded (Guo et al., 2016). For drought stress, seeds were grown on MS-medium containing 0, 100- and 200-mM mannitol and the root length was measured 8 days post sowing. Furthermore, 3-weeks-old *CaChiVI2*-overexpressed and wild-type *Arabidopsis* lines grown in soil-media were used for the analysis of heat and drought stress tolerance.

The *Arabidopsis* plants were incubated for a period of 16 h at 40°C for heat stress treatment. Plants were irrigated frequently during treatment to avoid drought stress. Furthermore, for drought stress, water was withheld from the seedlings for 5-days, while in control treatment, seedlings were provided with standard watering conditions. Leaf samples were collected for MDA, proline, and extraction of RNA. Randomly six separate seedlings were used for sample collection and then instantly frozen in liquid nitrogen and stored at −80°C. The experiment was conducted with three independent biological replicates for the accuracy of data.

### Physiological Attributes

To measure the contributing parameters of *CaChiVI2*-silenced pepper plants and *CaChiVI2*-overexpressed *Arabidopsis* plants, samples (0.5 g) were collected and finely grinded in liquid nitrogen. The malondialdehyde (MDA) content was measured through thiobarbituric acid (TBA) reaction method (De Vos et al., 1991). For this, 1.5 mL of the extract supernatant was mixed with 2 mL 0.6% (w/v) TBA solution dissolved in 5% (v/v) trichloroacetic acid (TCA) and heated in boiling water for 10 min. The supernatant was used for determination of MDA at 450 and 532 nm wavelength and subtracted from the absorbance at 600 nm. The activity of superoxide dismutase (SOD) was estimated by the inhibition of nitro-blue tetrazolium (NBT). The supernatant volume was illuminated at 4000 Lux for 20 min, then SOD activity was quantified spectrophotometrically at 560 nm. Control was determined in dark (Stewart and Bewley, 1980). Peroxidase (POD) activity was determined through guaiacol method (Bestwick et al., 1998). The reaction mixture used consisted of 50 mL 0.05 M phosphate buffer (pH7.8), 19 μL 30% H_2_O_2_ (v/v) and 28 μL guaiacol. The enzyme extract (0.5 mL) was added and a total of 3.0 mL of the reaction mixture was placed into a cuvette. The increase in absorbance was recorded at 470 nm at 30 s intervals for 3 min. Proline estimation was done according to Bates et al. (1973). A 2 ml of aqueous extract was mixed with 2 ml of glacial acetic acid and 2 ml of acid ninhydrin reagent (1.25 g of ninhydrin, 30 ml of glacial acetic acid, and 20 ml of 6 M orthophosphoric acid) and heated at 100°C for 30 min. After cooling, the reaction mixture was partitioned against toluene (4 ml) and the absorbance of the organic phase was firm at 520 nm. The resulting values were compared with a standard curve constructed using known amounts of proline (Sigma, St Louis, MO, United States).

Histochemical staining’s were performed to detect superoxide radical (O_2_^–^) and hydrogen peroxide (H_2_O_2_) in leaves. The leaves were inserted in 0.1% nitro-blue tetrazolium (NBT) with 50 mM potassium phosphate buffer (pH 7.8) for O_2_^–^ as described by Liu et al. (2012). For H_2_O_2_ detection, 3,3′-diaminobenzidine (DAB) solution was used in agroinfiltrated leaves. The leaves were incubated in 1.0 mg/mL DAB-HCl solution at room temperature, covered in dark for 12 h. Then de-stained by boiling in 95% ethanol for 5 min until the brown H_2_O_2_ spots on the leaves appeared (Thordal-Christensen et al., 1997; Kim et al., 2012). Relative electrolyte leakage (REL) was measured as per Feng et al. (2019), and the electrical conductivity percentage was calculated as REL (%) = C1/C2 × 100. For chlorophyll content, the readings were recorded on spectrophotometer after extracting into 80% (v/v) acetone (Arkus et al., 2005). Spectrophotometer (UV-1201 Shimadzu spectrophotometer, Japan) was used for all measurements.

### Statistical Analyses

IBM SPSS Statistics 25, United States were used for the statistical analysis. Significance differences between individual treatments were further analyzed at *P* ≤ 0.05 through Duncan Multiple Range (DMR) test. The analyzed data were expressed as mean and standard deviation (±SD). Three individual experiments were performed, and the data set for each biological replicate was used separately for analysis. The data were plotted by GraphPad Prism 8.0 (GraphPad Software, Inc., La Jolla, CA, United States).

## Results

### Cloning and *in silico* Analysis of Pepper *CaChiVI2*

**FIGURE 1:**
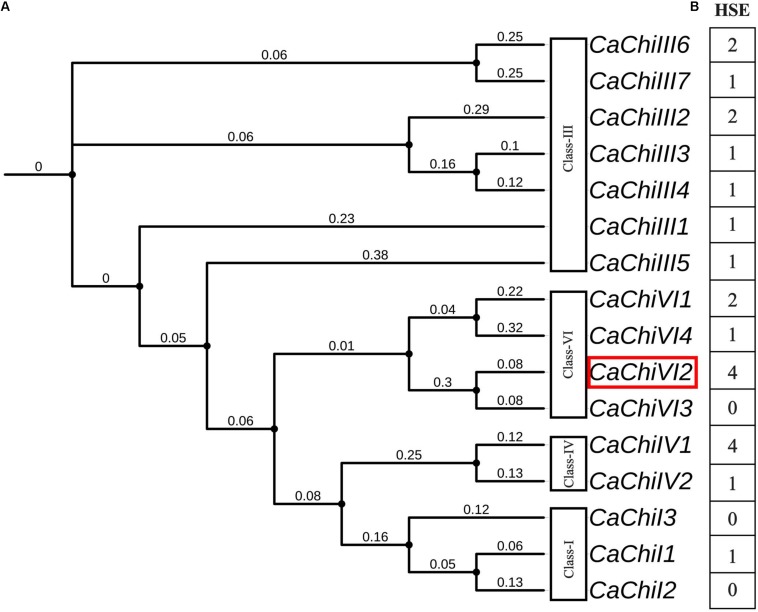
Phylogenetic relationship and *cis*-acting elements of CBP family genes in pepper. **(A)** Phylogeny of pepper CBP genes using iTOL (https://itol.embl.de/). The red color rectangle indicates the target gene **(B)** *Cis*-acting elements in the promoter regions of CBP genes inferred from the PlantCARE website (http://bioinformatics.psb.ugent.be/webtools/plantcare/html/). The highest number shows maximum, while the lowest shows minimum number of heat shock elements (HSE).

As previously reported, the evolutionary relationship between *CaChiVI2* and pepper CBP homologs in other species was based on phylogenetic analysis (Ali et al., 2018). The analysis revealed that pepper CBP could be classified into four main classes with potentially similar functions. Among all the chitin-binding protein (CBP) family genes, we identified a putative gene named *CaChiVI2* (Capana08g001237) (Figure 1A), which is re-derived from ‘Zunla’ database. The *CaChiVI2* was cloned using cDNA extracted from pepper leaves of AA3 pure-line. The full-length CDS of *CaChiVI2* cDNA consists of 606-bp and encodes 201 amino acids, while the genomic sequence contains 1203 nucleotides (Supplementary Table S6), including two exons and one intron (Ali et al., 2018). To investigate the possible *cis*-acting elements involved in the heat stimulation of defense-related genes, the 1.5 kb upstream region from the start codon (ATG) of all the CBP genes was analyzed with PlantCARE online server. The *in silico* analysis exhibited that heat stress elements (HSE) were available in the promoter region of 13 out of 16 members (Figure 1B) while the highest number (4) of HSE was found in *CaChiIV1* and *CaChiVI2*.

**FIGURE 2:**
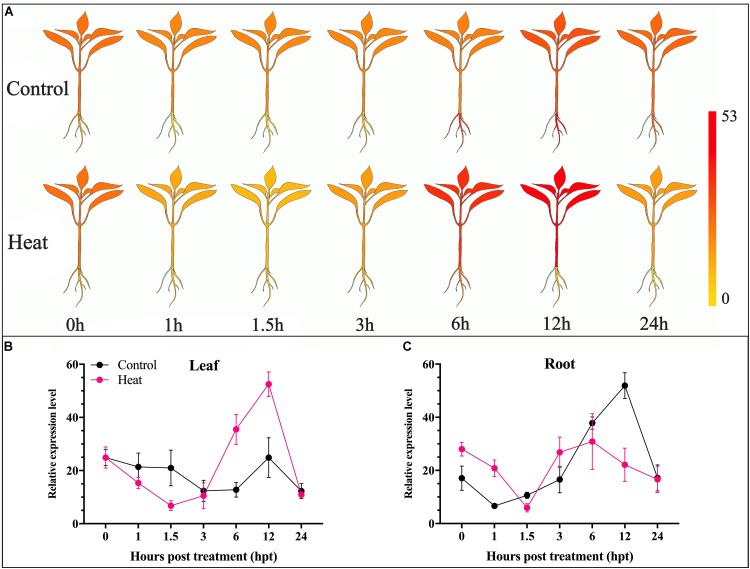
Transcriptomic analysis of *CaChiVI2* under heat stress. **(A)** The predicted expression level of *CaChiVI2* at different interval (0, 1.5, 3, 6, 12, and 24 h). Results were presented in heat map; the dark-yellow color represents strong down-regulation, and dark-red color represents strong up-regulation. **(B)** Transcriptomic results of *CaChiVI2* in the leaf of pepper plant under heat stress. **(C)** Transcriptomic results of *CaChiVI2* in the root of pepper plant under heat stress. These results were retrieved from Zunla database (http://pepperhub.hzau.edu.cn).

For further insight into the transcriptomic characteristics of *CaChiVI2* in pepper roots and leaves under heat stress, we initially practiced *in silico* analysis from a publicly available transcriptomic database of pepper (Zunla cultivar) (Liu F. et al., 2017). The diagrams display the index of a transcript ranging from yellow to red (Figure 2A). The transcript level of *CaChiVI2* in pepper revealed higher variance in distinct parts and at time intervals under heat stress, as demonstrated in Figures 2B,C. Moreover, the highest expression level (52.5) was noted in leaf tissue at 12 h interval followed by 35.5 at 6 h interval. In roots, the highest level (30.9) was recorded at 6 h interval. Overall, the *in silico* analysis revealed that expression in the leaf tissue was more elevated than the root.

### Protein Localization of *CaChiVI2*

**FIGURE 3:**
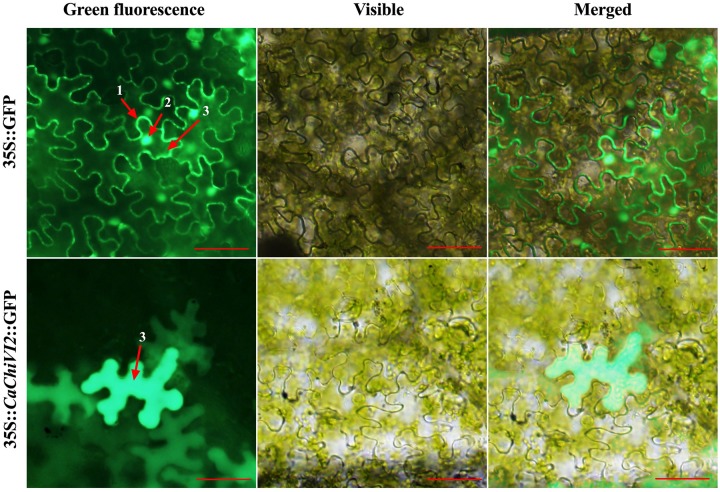
Protein localization of the pVBG2307:*CaChiVI2*:GFP in epidermal cells of tobacco leaves. pVBG2307:GFP was used as a control. The fluorescence was observed under a bright and fluorescent field. Every digit represents 1-cell membrane, 2-nucleus, and 3-cytoplasm, while red line indicate 100 μm.

The ORF fragment of *CaChiVI2* was recombined with the expression vector pVBG2307 that contained 35S promoter and reporter genes for green fluorescence protein (GFP). The pVBG2307:GFP and pVBG2307:*CaChiVI2*:GFP fused plasmids were infiltrated into *Nicotiana benthamiana* plants for *CaChiVI2* expression in epidermal tissue (Jin et al., 2019; Ma et al., 2019). The confocal laser micrographs exhibit that 35S:*CaChiVI2*:GFP fused protein were localized only in the cytoplasm of the cell (Figure 3). On the other side, the pVBG2307:GFP (mock) vector, used as a control, was give signal in three main parts of the cell including cell membrane, cytoplasm and nucleus.

### Expression Pattern of CBP Genes Under Heat Stress

**FIGURE 4:**
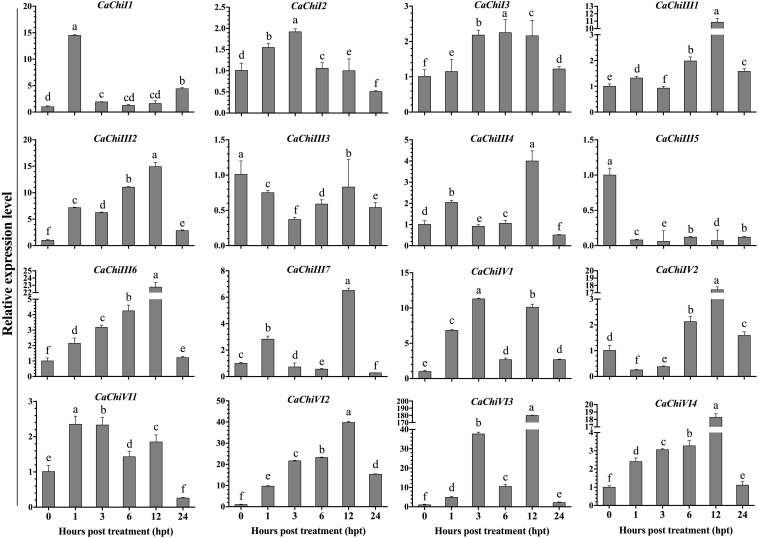
The transcript level of CBP genes in response to heat stress. The plant tissues were sampled at different time points (0, 1, 3, 6, 12, and 24 h post heat treatment) and were examined by qRT-PCR. The mean values and ± SDs for three replicates are presented. Different letters (a–f) on the bars in each histogram represent significant differences at *P* ≤ 0.05 using Duncan Multiple Range (DMR) test.

The seedlings of pepper plant were incubated at 45°C for heat stress to examine the transcript levels of CBP genes using qRT-PCR. The investigation revealed that different expression levels were found in different genes (Figure 4). Among all 16 genes, 14 were upregulated, while two genes (*CaChiIII3* and *CaChiIII5*) were downregulated at each time point. The *CaChiI1* initially expressed abruptly (14.5) at 1-h post-treatment then downregulated at all time points, whereas *CaChiI2*, *CaChiI3*, *CaChiIII2*, *CaChiIII6*, *CaVhiVI1*, *CaChiVI2*, and *CaChiVI4* progressively showed increased expression at each time point, till slight downregulation at 24 hpt. The *CaChiVI2* transcript level was the highest (39.76) at 12 hpt. Compared to other genes, its expression was significantly higher at every time point. Some members of CBP (*CaChiIII1*, *CaChiIII4*, *CaChiIV1*, and *CaChiVI3*) showed higher expression at some time points, though irregular changes were noticed in their expression level (Figure 4). Additionally, two genes *(CaChiIII7* and *CaChiIV2*) exhibited no remarkable changes in transcript levels against heat stress and their expression was not predominantly upregulated at various interval of time.

### *CaChiVI2*-Silencing Effect on Heat Stress Response and *P. capsici* Tolerance

**FIGURE 5:**
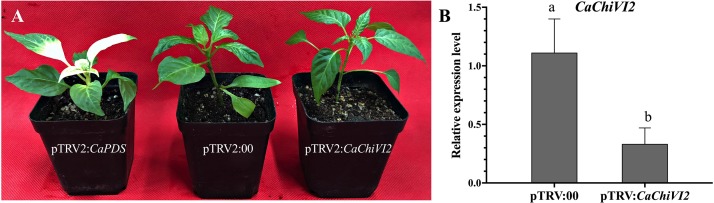
The phenotypes and silencing efficiency analysis of *CaChiVI2* pepper plants. **(A)** The phenotypes of TRV2:*CaPDS*, TRV2:00 and TRV2:*CaChiVI2.* **(B)** The silencing efficiency of *CaChiVI2* in the leaves of *CaChiVI2*-silenced and empty vector (TRV2:00) pepper plants. The bars show the mean values of three biological replicates and the error bars denote the standard deviation (+SD). Small alphabets (a–b) on the bars represent significant differences at *P* ≤ 0.05.

After 6-weeks of inoculation, the leaves of the positively controlled (pTRV2:*CaPDS*) plants exhibited photo-bleaching phenotype, which demonstrate the success of VIGS (Figure 5A). The silencing efficiency of pTRV2:*CaChiVI2* (*CaChiVI2*-silenced) and pTRV2:00 (control) plants were detected by qRT-PCR. As shown in Figure 5A, there was no visual difference between pTRV2:*CaChiVI2* and pTRV2:00 plants which grown in normal conditions and the silencing efficiency was around 71% (Figure 5B). Thus, *CaChiVI2*-silenced and controlled plants were used for further research.

**FIGURE 6:**
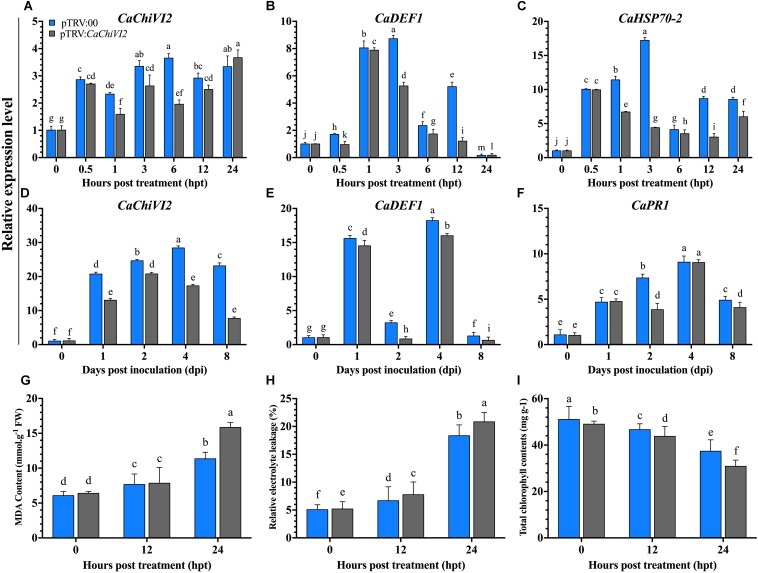
The *CaChiVI2*-silenced pepper plants tolerance to heat and *P. capsici* infection. **(A–C)** Transcript level of *CaChiVI2, CaDEF1*, and *CaHSP70-2* under heat stress, **(D–F)** transcript level of *CaChiVI2, CaDEF1*, and *CaPR1* during *P. capsici* inoculation. **(G)** MDA contents, **(H)** relative electrolyte-leakage, and **(I)** total chlorophyll content accumulation under heat stress. Mean values ± SDs for three replicates are shown. Different letters (a–m) in each individual histogram denote significant differences at *P* ≤ 0.05.

After heat stress treatment, the expression of *CaChiVI2* was checked in both pTRV2:*CaChiVI2* and pTRV2:00 plants. A remarkable difference was recorded in pTRV2:00 and pTRV2:*CaChiVI2* samples at all the time points, which revealed that the expression level of *CaChiVI2* was lower in pTRV2:*CaChiVI2* compared to pTRV2:00. A huge difference of >50% was found between pTRV2:*CaChiVI2* and pTRV2:00 at 6 h post-treatment (hpt) with values of 3.65 and 1.96 respectively (Figure 6A).

The regulation of other defense-related genes after the silencing of *CaChiVI2* showed that the transcript levels of resistance responsive genes, such as *CaHSP70-2* and *CaDEF1* (Do et al., 2004) increased in both *CaChiVI2*-silenced and control plants. Their transcript was significant lower in *CaChiVI2*-silenced plants comparatively control (TRV2:00) plants, for all the tested time points (Figures 6B,C). For examination of functional specificity of *CaChiVI2* under biotic stress, the TRV2:*CaChiVI2* (silenced) and TRV2:00 (controlled) pepper plants were infected with *P. capsici*. A higher transcript level of *CaChiVI2* was detected in control plants compared with *CaChiVI2*-silenced plants, with *CaChiVI2* expressing 66% more in pTRV2:00 plants. As shown in Figure 6D, the silencing of *CaChiVI2*-gene had a significant tolerance role against *P. capsici*.

We further explored the expression levels of other stress responsive genes to find the interaction/role of *CaChiVI2* with other resistance responsive genes by altering their expression. It was noted that after *P. capsici* inoculation, *CaDEF1* (Do et al., 2004) and *CaPR1* gave a positive response. Their transcript level in the pTRV2:00 plants was better than that of the TRV2:*CaChiVI2* plants at all tested time points (Figures 6E,F).

**FIGURE 7:**
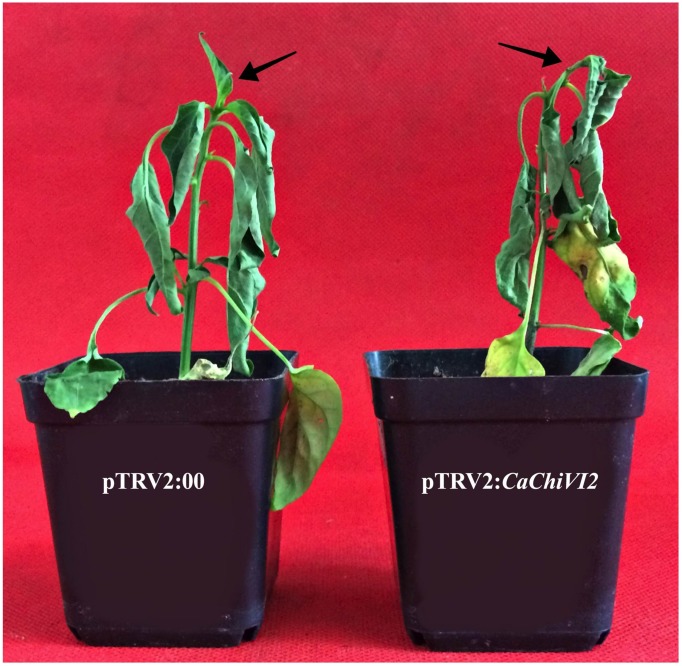
Phenotypes, heat stress recovery assay of the *CaChiVI2*-silenced (pTRV2:*CaChiVI2*) and control (pTRV2:00) plants after 5-days at 22°C.

After heat stress treatment, the MDA contents and REL in *CaChiVI2*-silenced plants were significantly higher than control plants (Figures 6G,H). More severe symptoms of wilting and yellowing were observed in *CaChiVI2*-silenced plants, which indicated that chlorophyll contents degraded more in pTRV2:*CaChiVI2* plants (Figure 6I). Moreover, the recovery efficiency was also checked which revealed that pTRV2:00 plants recovered their growth quicker than TRV2:*CaChiVI2* plants, when incubated at normal temperature (22°C) (Figure 7).

**FIGURE 8:**
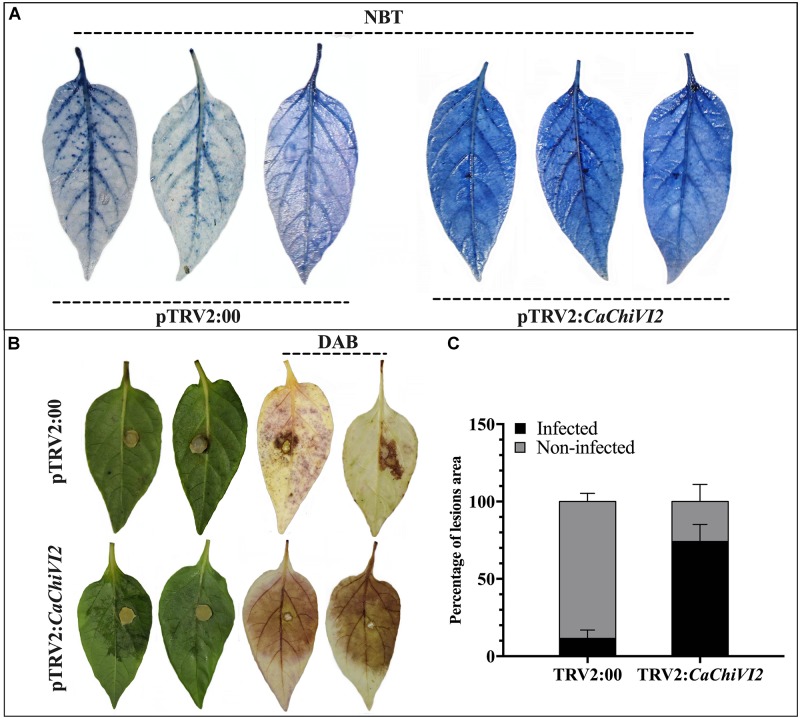
Detection of O_2_^–^ and H_2_O_2_ concentration in *CaChiVI2*-silenced and control pepper plants. **(A)** Heat stress symptoms appearance on detached leaves of TRV2:*CaChiVI2* and TRV2:00 pepper plants, NBT staining shows detection of O_2_**^–^** concentration. **(B)** *P. capsici* symptoms (lesions) developed on pepper leaves of TRV2:*CaChiVI2* and TRV2:00 plants, DAB staining shows detection of H_2_O_2_. **(C)** Percentage of the infected and non-infected areas of pepper leaves after *P. capsici* infection. The bars represent the means ± SD of three independent biological replicates.

Production of ROS is a typical consequence once plants are in stress condition. We noticed a higher accumulation of superoxide (O_2_^–^) and hydrogen peroxide (H_2_O_2_) using NBT and DAB staining respectively. After heat stress, a higher production of O_2_^–^ was detected in *CaChiVI2*-silenced plants, leaves (dark-blue color) compared with control plants (light-blue color) (Figure 8A). After 3 days of inculcation, the *Phytophthora capsici* lesions were detected on the isolated leaves of both TRV2:*CaChiVI2* and TRV2:00 pepper plants. However, the infected areas and H_2_O_2_ accumulation were more pronounced in silenced plant versus control (Figure 8B). Quantitative analysis exhibited that disease infected area of TRV2:*CaChiVI2* plants (>74%) was significantly more expanded than the TRV2:00 plants (11.6%) (Figure 8C).

### Overexpression of *CaChiVI2* Improve the Tolerance Against Heat Stress Damage in *Arabidopsis*

**FIGURE 9:**
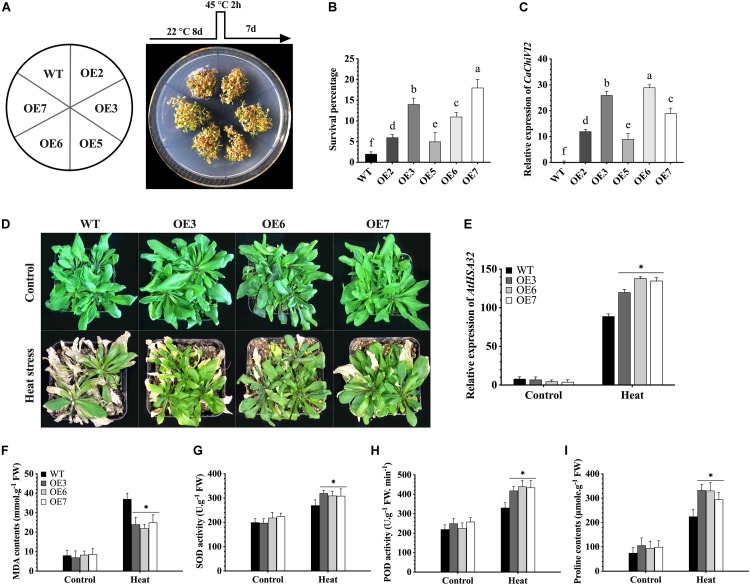
*CaChiVI2*-overexpression effect on heat stress tolerance in transgenic *Arabidopsis* lines. **(A)** *CaChiVI2*-overexpressed *Arabidopsis* lines (OE2, OE3, OE5, OE6, and OE7) and WT under heat stress. The different heat stress regimes as schematically shown on the top of petri-dish. **(B)** Comparison of *CaChiVI2*-overexpressed *Arabidopsis* lines (OE2, OE3, OE5, OE6, and OE7) and WT. Survival percentage of 8-day-old seedlings after exposing to 45°C heat stress for 2 h and then keeping at 22°C for 7 days. **(C)** The relative expression profile of *CaChiVI2* under normal condition in *CaChiVI2*-overexpressed and WT plants. **(D-I)** Phenotypes, the transcript level of *AtHSA32*, MDA contents, SOD, POD activity and Proline accumulation measured in transgenic lines (OE3, OE6, and OE7) and WT plants after 40°C heat stress treatment for 16 h. *Arabidopsis* seedlings grown at 22°C were used as control. The bars indicated the mean values ± SDs for three replicates. The small letters (a–m) and asterisk (*) in corresponding graphs denote significant differences at *P* ≤ 0.05.

In order to select the best thermo-tolerant homozygous T_3_ lines for further analysis, wild-type (WT) and five *CaChiVI2*-overexpressed (OE2, OE3, OE5, OE6, and OE7) *Arabidopsis* lines were cultured on MS-media and were kept at 45°C (high temperature) for 2h. The *CaChiVI2-*overexpressed plants were then kept at 22°C for 7-days to recover and their survival rate was measured. The results exhibited that the survival rate of OE3, OE6, and OE7 lines was higher than WT, OE2, and OE5-lines (Figures 9A,B). Besides, some WT and transgenic *Arabidopsis* lines were grown in normal environmental conditions to detect the transcript levels of *CaChiVI2* gene. As presented in Figure 9C, the transcript levels of *CaChiVI2* in OE3, OE6, and OE7 lines were significantly higher than the WT seedlings. Thus, the OE3, OE6, and OE7 were selected for further studies.

The seedlings were then grown in composite soil and treated with 40°C temperature for 16 h. As a result, the leaves of WT seedlings wilted severely, while mildly in case of *CaChiVI2*-OE seedlings. Ten days after heat treatment, the WT did not show progressive growth as compared to transgenic lines, as shown in Figure 9D. However, the defense-related gene (*AtHSA32*) was highly expressed in both WT and transgenic *Arabidopsis*. Additionally, the expression level of *AtHSA32* in transgenic-lines (OE3, OE6, and OE7) was substantially higher than WT plants (see Figure 9E). Moreover, the accumulation of MDA contents in *CaChiVI2*-overexpressed lines was lower (Figure 9F), while SOD, POD activity and proline contents of *CaChiVI2*-OE seedlings were higher as compared to WT (Figures 9G–I).

### Overexpression of *CaChiVI2* Enhances Endurance to Drought Stress in *Arabidopsis*

**FIGURE 10:**
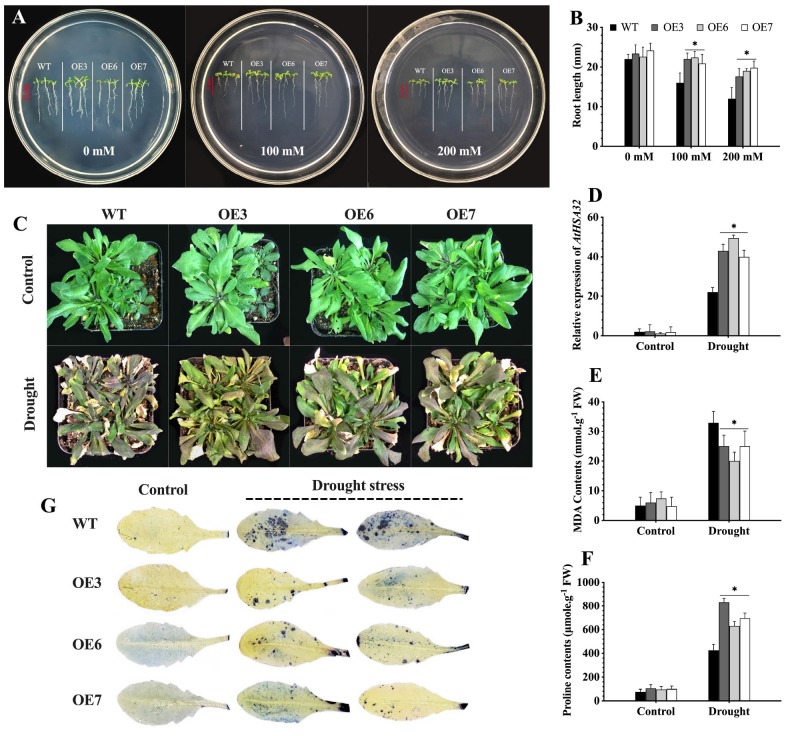
*CaChiVI2*-overexpression effect on drought stress tolerance of transgenic *Arabidopsis*. **(A)** Root growth (length) of *CaChiVI2*-overexpressed lines (OE3, OE6, and OE7) and WT *Arabidopsis* seedlings grown for 8-days on MS-medium containing 0, 100, and 200 mM mannitol. **(B)** Root length of the seedlings. **(C–F)** Phenotypes, the transcript level of *AtHSA32*, MDA contents, and proline accumulation of transgenic lines (OE3, OE6, and OE7) and WT plants under 5 days drought stress treatment (control plants were watered regularly). **(G)** Concentration of O_2_**^–^** (superoxide) in the leaves of transgenic lines (OE3, OE6, and OE7) and WT *Arabidopsis* plants after drought stress treatment (NBT staining technique was used for O_2_**^–^** detection). Bars in the respective histograms are mean values of three replicates and error bars are ± SDs. Asterisk (*) denotes significant differences at *P* ≤ 0.05.

The *CaChiVI2* overexpressed transgenic lines were investigated for drought stress tolerance on MS-medium containing 0, 100, and 200 mM mannitol. There were no differences in the germination rates of WT plants and transgenic lines after 5 days. However, the root length of *CaChiVI2*-OE lines was substantially longer than the WT seedlings (Figures 10A,B). Besides, the WT and transgenic seedlings were grown in soil-media where water was withheld for 5 days. As a result, the WT seedlings dehydrated and displayed severe wilting. In comparison, slight damage was observed in transgenic seedlings and the rate of recovery was also faster than WT plants (Figure 10C). Nevertheless, the drought stress-related gene *AtHSA32* was elicited in both transgenic and WT *Arabidopsis*, however its transcript level was greater in transgenic seedlings compared to wild-type plants (Figure 10D). Besides, the MDA content was substantially lower and proline significantly higher in *CaChiVI2*-OE seedlings as compared to WT (Figures 10E,F). Additionally, the accumulation of O_2_^–^ was lower in transgenic seedlings (Figure 10G).

## Discussion

The CBP is a stress-responsive multigene family, which regulates resistance against biotic and abiotic stresses (Xu et al., 2007; Ahmed et al., 2012; Su et al., 2015). The specificity of CBP-family regarding various stresses is confirmed in wheat crop (Singh et al., 2007). However, study on the characterization of pepper CBP genes, especially in relation to heat and drought stress is still not conclusive. Therefore, to fill the gap and disclose more CBP functions, *CaChiVI2* gene from the *Capsicum annuum* L. genome database was chosen for further analysis. Likewise other CBP members, *CaChiVI2* also had four heat shock elements (HSE’s) in their promoter region (Pastuglia et al., 1997; Ali et al., 2018). To confirm its function, the *CaChiVI2* in pepper was successfully knockdown by VIGS, which correspondingly reduced resistance against heat stress and *P. capsici* infection (Figures 6A,D). Additionally, *CaChiVI2* knockdown affect the expression of other stress responsive genes, i.e., *CaDEF1* and *CaHSP70-2* under heat stress and *CaPR1* and *CaDEF1* against *P. capsici* infection, which showed its role in defense mechanism of pepper plant. This statement is supported by Jin et al. (2016) through studying the function of *CaPTI1* in pepper plant. The findings indicate that *CaChiVI2* has a crucial role in enhancing pepper plants endurance against high temperature and *P. capsici* infection.

Reactive oxygen species signal components such as H_2_O_2_ and O_2_^–^ play a significant role in plants responses to unfavorable environmental conditions. They perform multiple roles by acting directly in the initial defense. However, the over-accumulation of H_2_O_2_ and O_2_^–^ under stress results in an active damage to the cell structures, photosynthesis and natural intracellular environment (Uzilday et al., 2012; Tuteja et al., 2014; Srivastava et al., 2016; Haq et al., 2019). Therefore, the contents of H_2_O_2_ and O_2_^–^ are often used to find the damage levels of plant cells (Li et al., 2018). The detached leaves assay of *CaChiVI2*-silenced and control (TRV2:00) plants after the heat and *P. capsici* treatments were established to assess the ROS activities. As a result, more infection and ROS accumulation were found in the leaves of *CaChiVI2*-silenced versus control, which exhibited that knockdown of *CaChiVI2* increased sensitivity and plants became more prone to stresses (Figures 8A,B). The outcome followed the similar pattern as explained in previous study by Zhang et al. (2018), and our conclusion are also consistent with Su et al. (2015). Contrarily, when *CaChiVI2* overexpressed in *Arabidopsis*, low O_2_^–^ accumulation was recorded in overexpressed plants as compared to WT under drought stress (Figure 10G). This indicates the role of *CaChiVI2* in the defense system of a plant and also suggests that *CaChiVI2* confers the plants ability to reduce the H_2_O_2_ and O_2_**^–^** concentrations thus evading the injuries caused by ROS under heat, drought and *P. capsici* infection.

Previously, overexpressed tobacco plants confirmed resistance to high temperature and oxidative stress exhibiting higher physiological indicators than WT plants (Zhang J. et al., 2016; Zang et al., 2018) and higher survival rate and root length was observed under high temperature and NaCl stress (Li J. et al., 2016). *CaChiVI2* overexpression showed moderate heat and drought stress symptoms and increased the resistance of *Arabidopsis* plants (Figures 9D, 10C). The transcriptional activation/up-regulation of *CaChiVI2* is necessary for immediate resistance against heat in *CaChiVI2*-OE lines (Figure 9C), signifying that *CaChiVI2* not only responds to heat stress but also participates in drought stress. These facts confirm the earlier conclusions that CBP genes play an important role in environmental stresses (Hamid et al., 2013). Furthermore, resistance to high temperature and drought stress is also regulated by other genes in *Arabidopsis*, such as acquired thermo-tolerance related gene *AtHSA32* is strongly activated by heat stress (Charng et al., 2006; Burke and Chen, 2015; Iurlaro et al., 2016). In our study, the transcript level of *AtHSA32* gene was highly provoked by high temperature and drought stress in *CaChiVI2*-OE *Arabidopsis* as compared to WT plants (Figure 9E). It suggests that *CaChiVI2* may possibly be involved in heat stress endurance by altering the transcript level of stress responsive genes. However, further study is needed to understand the exact mechanism.

Malondialdehyde (MDA) is an important product of membrane lipid peroxidation (LPO), which is considered as a reliable biochemical oxidative stress marker (Del Rio et al., 1996; Koźmińska et al., 2019). Based on our findings, it can be determined that the *CaChiVI2-*silenced plants exhibited an increase in MDA level, though the *CaChiVI2*-overexpressed *Arabidopsis* plants showed a decrease in MDA level (Figures 6G, 9F, 10E). The oxidative stress has shown correlation with MDA level (Gechev et al., 2002; Madhusudhan et al., 2009). It suggests that this might be due to the silencing effect of *CaChiVI2* leading to damage in the plasma membrane. Whereas, the *CaChiVI2* overexpressed *Arabidopsis* plants tolerate heat and the plasma membrane damages due to drought stress.

To mitigate the damages of ROS in unfavorable environmental conditions, plants develop special mechanisms to scavenge glut ROS through antioxidant enzymes such as SOD, POD, CAT, and APX and non-enzymatic antioxidants (Vitamin-C and proline content). These antioxidant enzymes and proline reduce oxidative damage caused by stress (Schöffl et al., 1999; Feng et al., 2019). During the scavenging of ROS, the SOD first decomposes O_2_^–^ to H_2_O_2_ and then the H_2_O_2_ is scavenged by peroxidase (POD) in the cytosol and the outer cellular space (Gill and Tuteja, 2010; Uzilday et al., 2012). It is also investigated that the CBD having cysteine and the hinge region, which is saturated by proline and glycine. The biosynthesis of proline decreases the injury caused by ROS (Schöffl et al., 1999), while in many plant species proline has been considered one of the most common compatible osmolyte for cellular osmotic adjustment which are conferred by the high salinity, water deficit and other stresses (Szabados and Savoure, 2010; Al Hassan et al., 2017; Kumar et al., 2017). Likewise, in our study, the *CaChiVI2*-overexpressed *Arabidopsis* revealed significantly higher SOD, POD activities and proline contents than WT plants under heat stress (Figures 9G–I, 10F). Therefore, the previous findings of Sun et al. (2019), where transgenic *Arabidopsis* revealed better resistance to high temperature by accumulating more CAT, SOD and POD activity compared to wild-type plants, support our results. Interestingly in our study, the observation of *CaChiVI2*-OE lines showed higher proline biosynthesis relative to WT plants under heat and drought stress conditions (Figures 9I, 10F). Therefore, it can be concluded that the regulation of *CaChiVI2* under CaMV35S promoter is clearly overexpressed and significantly influences the antioxidant enzymes machinery and also proline biosynthesis in response to heat and drought stress. In plants the electron transport chain regulated by mitochondrial second terminal oxidase (alternative oxidase-AOX) is also vital for the defensive machinery of a plant (Dong et al., 2009). Keeping in view this theory, we may conclude that the overexpression of *CaChiVI2* upregulates the expression of AOX gene and thereby participates in the scavenging of ROS pathway and improves the overall defense system. Our findings clearly demonstrate that *CaChiVI2* may tolerate high temperature and drought condition through the ROS-scavenging pathway. However, before declaring the potential role of *CaChiVI2* as a novel stress-responsive gene, it is required to evaluate the exact mechanisms and regulation under heat signaling pathways.

The subcellular localization of CBP members may show their associated functions. Some CBP genes in other plants such as Sc*ChiI1* have been localized in the cytoplasm and the plasma membrane (Su et al., 2015) and *ChiIV3* in the plasma membrane (Liu Z. et al., 2017). In *Oryza sativa*, CBP proteins are located in cytoplasm and mitochondria and are thought to be associated with the thylakoid membrane under heat stress (Xu et al., 2007). The transient expression of *CaChiVI2* in *N. benthamiana* leaves confirmed the localization of *CaChiVI2* proteins only in the cytoplasm, indicating a vital role in the cytoplasm of a cell (Figure 3). Our key findings from testing the cloned *CaChiVI2* gene are that heat stress and subsequently silencing by VIGS, caused severe damages to plant morphology and physiology. The ectopic expression of *CaChiVI2* in *Arabidopsis thaliana* showed lethal damage due to heat and drought condition by increasing the transcript of resistance responsive genes. In the light of previous studies, we can conclude that *CaChiVI2* may be acting as a transcriptional activator and provide an innate response to high temperature and drought condition.

## Conclusion

Our results validated that *CaChiVI2* knockdown decreased the stress tolerance ability of pepper plants against heat and *P. capsici* infection; additionally, the physiological and morphological attributes were also altered at certain level. The co-regulatory response of other defensive genes such as *CaDEF1, CaPR1*, and *CaHSA70-2* not only confirmed the functional diversity of *CaChiVI2* gene, but also introduce the important role in stress tolerance mechanism. In contrast, *CaChiVI2*-overexpressed plants showed mild symptoms, decreased heat and drought stress with the elevated transcript levels of the defense-related gene, including *AtHSA32.* The identified tasks of *CaChiVI2* gene may provide some solid proofs for advance study of pepper plant adaptation mechanisms in response to sever environmental conditions.

## Data Availability Statement

All datasets generated for this study are included in the article/Supplementary Material.

## Ethics Statement

The study presented in the manuscript did not involve any experimentation on human or animal subjects.

## Author Contributions

MHA and Z-HG conceived and designed the research. MKA conducted the experiments and wrote the manuscript. KA and IM analyzed the data. SH and AK performed the *in silico* analysis. AK, MHA, and HU critically revised the manuscript. Z-HG and GL contributed reagents and funded the project. All the authors read and approved the manuscript.

## Conflict of Interest

The authors declare that the research was conducted in the absence of any commercial or financial relationships that could be construed as a potential conflict of interest.

## Footnotes

1 https://www.genome.jp/tools-bin/clustalw

2 https://itol.embl.de/

3 http://bioinformatics.psb.ugent.be/webtools/plantcare/html/

4 http://pepperhub.hzau.edu.cn
