# Supplementary material for: The CaChiVI2 Gene of Capsicum annuum L. Confers Resistance Against Heat Stress and Infection of Phytophthora capsici
Source: Front Plant Sci. 2020 Feb 26;11:219. doi: 10.3389/fpls.2020.00219 (PMC7057250; doi:10.3389/fpls.2020.00219)
Supplement: Supplementary file 1 [file Table_1.DOCX]

**The *CaChiVI2* gene of *Capsicum annuum* L. confers resistance against heat stress and infection of *Phytophthora capsici***

Muhammad Ali^1,2^, Izhar Muhammad^3^, Saeed ul haq^1^, Mukhtar Alam^4^, Abdul Mateen Khattak^5^, Kashif Akhtar^6^, Hidayat Ullah^4^, Abid Khan^1^, Gang Lu^2,^*, Zhen-Hui Gong^1,^*

^1^College of Horticulture, Northwest A&F University, Yangling, Shaanxi, 712100, P. R. China

^2^Department of Horticulture, Zhejiang University, Hangzhou 310058, P. R. China

^3^College of Agronomy, Northwest A&F University, Yangling 712100, P. R. China

^4^Department of Agriculture, The University of Swabi, Khyber Pakhtunkhwa, Pakistan

^5^Department of Horticulture, The University Agriculture, Peshawar, Khyber Pakhtunkhwa, Pakistan

^6^Institute of Nuclear Agricultural Sciences, College of Agriculture and Biotechnology, Zhejiang University, Hangzhou 310058, China

^*^Corresponding authors: Zhen-Hui Gong, E-mail: [zhgong@nwsuaf.edu.cn](mailto:zhgong@nwsuaf.edu.cn); Gang Lu, E-mail: [glu@zju.edu.cn](mailto:glu@zju.edu.cn)

**Table S1. The CDS sequence of *CaChiVI2* (Accession no. Capana08g001237)**

| CDS sequence | Protein sequence |
| --- | --- |
| ATGGAGAAGCTAAGTACTACTGCTCTTTTGCTGTCTTTGGTCCTCTTCATCATAGCCGCAGTTGCAAACGCACAACAGTGTGGGAGGCAAAGGGGCGGAGCGGTATGCAGCGGGAGCTTGTGTTGCAGCCAGTATGGTTGGTGTGGATCGACACCCGAATACTGTTCACCTAGCCAAGGTTGTCAGAGCCAATGCGGTGGCAGTGTACCAACTCCAACTCCAGGAGGAGGTGGGGCTAGCGCGCAAAATGTACGTGCAACATATCATTTGTATAACCCGCAGAATGTTGGGTGGGACTTGAATGCGGTTAGTGCTTATTGCTCTACTTGGGATGCTAATAAGCCTTTGGCCTGGAGGAGCAAGTATGGTTGGACTGCTTTCTGTGGTCCTGTTGGACCTCGTGGTCGAGACTCATGCGGCAAGTGCTTAAGGGTGACAAACACACGCACAGGAGCTCAGACGATAGTGAGAATCGTGGATCAATGCAGCAATGGTGGACTAGATTTAGACGTTAACGTTTTCCGTCAAATCGATACGGACGGAGTAGGAAATCAACGAGGCCACCTTATTGTGAACTACCAGTTTGTTGATTGTGGTGATAACTGA | MEKLSTTALLLSLVLFIIAAVANAQQCGRQRGGAVCSGSLCCSQYGWCGSTPEYCSPSQGCQSQCGGSVPTPTPGGGGASAQNVRATYHLYNPQNVGWDLNAVSAYCSTWDANKPLAWRSKYGWTAFCGPVGPRGRDSCGKCLRVTNTRTGAQTIVRIVDQCSNGGLDLDVNVFRQIDTDGVGNQRGHLIVNYQFVDCGDN |

**Table S2. Primers for qRT-PCR of chitin-binding protein family genes of pepper.**

| Gene name | RT-PCR Primer sequence (5'→3') | Product length |
| --- | --- | --- |
| *CaChiI1* | F: AAACCTCCCATGAAACTACCG | 227 |
|  | R: GTTTAAAAGGTCAACTCCGATGG |  |
| *CaChiI2* | F: GGACATCAGCGGTGTAATCT | 116 |
|  | R: CAGCATTGATGAAGGCATTGT |  |
| *CaChiI3* | F: CTATTTGCTCTCTTATTGCTGATGG | 133 |
|  | R: CAGGACCACAATAGGCATCG |  |
| *CaChiIII1* | F: TCTTCCCTTTCATCTTCCAACC | 229 |
|  | R: AGTTTGTTCTCCGCATCGTC |  |
| *CaChiIII2* | F: CTCCACCTCCACCTAATTTCC | 100 |
|  | R: CCACACCAACCCCATCTAC |  |
| *CaChiIII3* | F: TGGTGCGGAACTACAGAAAAG | 127 |
|  | R: TTCTACCATCAGCTTGCCTTC |  |
| *CaChiIII4* | F: GGAATGCAAGCTGATGGTAGA | 110 |
|  | R: CTCTGACTTTGACACCACTCTT |  |
| *CaChiIII5* | F: GAAAGCAAGCTGCTGGTAGA | 106 |
|  | R: TGACTTTGACAGTAGGGAGAAC |  |
| *CaChiIII6* | F: GTCCTAGTGAAGAGTGTTGTAGTT | 114 |
|  | R: CGCATAAACCTGGCTCTTCT |  |
| *CaChiIII7* | F: TCAGCCAAAACATCTCTTCCC | 121 |
|  | R: CATCAGGGCATTCTCTACCAC |  |
| *CaChiIV1* | F: TCTTTGCTCATGTCACCCAC | 227 |
|  | R: ATCTCTTGCAACTATGTCAGGG |  |
| *CaChiIV2* | F: TCAAGTGCTATCCTGAATTCGG | 182 |
|  | R: CTTTCCAGAGACACAAGGGTAC |  |
| *CaChiVI1* | F: CAATACGGGTTCTGTGGTACG | 103 |
|  | R: CAACATTTTCAGCTTCGCCAG |  |
| *CaChiVI2* | F: TGGGACTTGAATGCGGTTAG | 178 |
|  | R: TCACTATCGTCTGAGCTCCTG |  |
| *CaChiVI3* | F: CGACATGGGATGCTAATAAGCC | 143 |
|  | R: CGTTGTTTGAGCTCTGGTTCG |  |
| *CaChiVI4* | F: GTCTTTGTGATTTTGGCCCTG | 191 |
|  | R: TTGGCGAACATGGTAGTGG |  |

**Table S3. Primers for subcellular localization of *CaChiVI2***

| **Primer Name** | **Primer sequence (5'→3')** | **Enzymes** |
| --- | --- | --- |
| *CaChiVI2* | F: GCTCTAGATGAAACAACATCAATCAAAT | XbaI |
|  | R: GGGGTACC5GTTATCACCACAATCAACAA | Kpn1 |

**Table S4. Primers for gene silencing of *CaChiVI2***

| **Primer Name** | **Primer sequence (5'→3')** | **Enzymes** |
| --- | --- | --- |
| *CaChiVI2* | F: CCGGAATTCACTGCTTTCTGTGGTCCTGT | EcoR1 |
|  | R: CCGCTCGAGTTATCACCACAATCAACAAACTG | Xho1 |

**Table S5. Primers for overexpression of *CaChiVI2***

| **Primer Name** | **Primer sequence (5'→3')** | **Enzymes** |
| --- | --- | --- |
| *CaChiVI2* | F: GCTCTAGATGAAACAACATCAATCAAAT | XbaI |
|  | R: GGGGTACC5GTTATCACCACAATCAACAA | Kpn1 |

**Table S6. The genomic sequence of *CaChiVI2***

ATGGAGAAGCTAAGTACTACTGCTCTTTTGCTGTCTTTGGTCCTCTTCATCATAGCCGCAGTTGCAAACGCACAACAGTGTGGGAGGCAAAGGGGCGGAGCGGTATGCAGCGGGAGCTTGTGTTGCAGCCAGTATGGTTGGTGTGGATCGACACCCGAATACTGTTCACCTAGCCAAGGTTGTCAGAGCCAATGCGGTGGCAGTGTACCAACTCCAACTCCAGGAGGAGGTGGGGCTAGCGCGCAAAATGTACGTGCAACATATCATTTGTATAACCCGCAGAATGTTGGGTGGGACTTGAATGCGGTTAGTGCTTATTGCTCTACTTGGGATGCTAATAAGCCTTTGGCCTGGAGGAGCAAGTATGGTTGGACTGCTTTCTGTGGTCCTGTTGGACCTCGTGGTCGAGACTCATGCGGCAAGTGCTTAAGGGTAAGATATATGCATGGACTACTCTATTTTTTATGATAACATTTCAATAATTACTACCGTCTTCTATGATAACATTTCATTATGTTACATTAGATTAGATCGATGCATGTTCAATATAATACTGGCTCCGTTTTAAATTTGTTTGGCCTACTTTCATTTTCAGTCTATTTCACAAAGTAAGACCTTTTAATCTCAACCTTCTACATGACATTTTTAGAGGCCACACGCGCGGTTAAAGAACATTTTGGTTCATTTGATATATCTTGAATTTAATATCAAATGATTCAAAAGTTTATATACTTTTTTAACTTTGTGCTAAGTCAACATATATAGGTCAGATAAATTGAAACGAAGAAAGTAACACAATTTGATATAGCAGTAAAAAAATATCAGGGTGGCTATATTATATAGAGATTTGTTTTGTGGCAGTTTATGTCACAGAATACACCCTAAAATTCTGCATTTTGAATGATCATCGACTTTTGGAGGTCAAAATTATTACTACTATATGTTTAGTACGTATTTCATTAGTTTCTAACTATCTAATATTTAACTCATGAACTGACACCATCACAAATTACATATATATATGTACAGGTGACAAACACACGCACAGGAGCTCAGACGATAGTGAGAATCGTGGATCAATGCAGCAATGGTGGACTAGATTTAGACGTTAACGTTTTCCGTCAACTCGATACGGACGGAGTAGGAAATCAACAAGGCCACCTTATTGTGAACTACCAGTTTGTTGATTGTGGTGATAACTGA
